# Rybp, a polycomb complex-associated protein, is required for mouse eye development

**DOI:** 10.1186/1471-213X-7-39

**Published:** 2007-04-30

**Authors:** Melinda K Pirity, Wei-Lin Wang, Louise V Wolf, Ernst R Tamm, Nicole Schreiber-Agus, Ales Cvekl

**Affiliations:** 1 Department of Molecular Genetics, Albert Einstein College of Medicine, Bronx, NY 10461, USA; 2 Institute of Human Anatomy and Embryology, University of Regensburg, Regensburg, Germany; 3 Ophthalmology and Visual Sciences, Albert Einstein College of Medicine, Bronx, NY 10461, USA

## Abstract

**Background:**

Rybp (Ring1 and YY1 binding protein) is a zinc finger protein which interacts with the members of the mammalian polycomb complexes. Previously we have shown that Rybp is critical for early embryogenesis and that haploinsufficiency of *Rybp *in a subset of embryos causes failure of neural tube closure. Here we investigated the requirement for *Rybp *in ocular development using four *in vivo *mouse models which resulted in either the ablation or overexpression of *Rybp*.

**Results:**

Our results demonstrate that loss of a single *Rybp *allele in conventional knockout mice often resulted in retinal coloboma, an incomplete closure of the optic fissure, characterized by perturbed localization of *Pax6 *but not of *Pax2*. In addition, about one half of *Rybp-/- <-> Rybp+/+ *chimeric embryos also developed retinal colobomas and malformed lenses. Tissue-specific transgenic overexpression of *Rybp *in the lens resulted in abnormal fiber cell differentiation and severe lens opacification with increased levels of *AP-2α *and *Sox2*, and reduced levels of *βA4-crystallin *gene expression. Ubiquitous transgenic overexpression of *Rybp *in the entire eye caused abnormal retinal folds, corneal neovascularization, and lens opacification. Additional changes included defects in anterior eye development.

**Conclusion:**

These studies establish *Rybp *as a novel gene that has been associated with coloboma. Other genes linked to coloboma encode various classes of transcription factors such as *BCOR*, *CBP*, *Chx10*, *Pax2*, *Pax6*, *Six3*, *Ski*, *Vax1 *and *Vax2*. We propose that the multiple functions for *Rybp *in regulating mouse retinal and lens development are mediated by genetic, epigenetic and physical interactions between these genes and proteins.

## Background

The vertebrate eye is a complex neurosensory organ. Normal function of the eye requires precise spatial organization and interaction between individual tissues to respect the laws of optics. During embryonic development, reciprocal inducing events result in the formation of the lens and the retina that originate from progenitor cells located in the head surface ectoderm and neuroepithelium of the ventral diencephalon, respectively (see reviews:[1,2]). Abnormal lens and retinal development can cause isolated or widespread ocular abnormalities that can obstruct vision at different levels and lead to blindness (reviewed in [3]).

Between embryonic day E9.5 and E11.5 of mouse development, the optic vesicle undergoes dorso-ventral patterning of the neuroepithelium followed by its invagination to form the bilayered optic cup [4]. This process gives rise to the optic fissure, allowing blood vessels originating from the vascular mesoderm to enter the developing eye. By E13.5, the nasal and temporal retina on either side of the choroid fissures fuses around the optic nerve axons and blood vessels. Failure of this closure results in a specific developmental abnormality, called ocular coloboma. Ocular coloboma is often seen in association with severe neurological and/or craniofacial abnormalities [5] or can develop as an isolated condition [6].

Lens differentiation at E12.5 is marked by cellular elongation of the lens cells forming the posterior part of the lens vesicle [7]. Differentiating lens fiber cells upregulate expression of various classes of structural proteins including crystallins, intermediate filament bead proteins, and cytoskeletal, membrane, and channel proteins. Abnormal lens fiber cell differentiation disrupts lens homeostasis leading to precipitation of lens proteins resulting in lens opacification and perturbed vision (see review [8]).

At the molecular level, a significant number of genes involved in the control of eye development also regulate brain development [9]. The most notable classes are homeobox genes such *as Lhx2*, *Otx2*, *Pax6*, *Rx and Six3*, and the basic helix-loop-helix genes *Math5*, *Neurod1 *and *Neurog2*. In contrast, other genes play specialized roles during lens (e.g., *Foxe3*, *Mab21like1*, *c-Maf*, *Pitx3*, *Sox1 and Hsf4*) or retinal (e.g., *Chx10*, *Mab21like2*, *Six6/Optx2*, *Vax1*, *Vax2*) development [10,11]. Several global regulatory genes are also integral to normal ocular development. For example, *Brg1 *[12], *Snf2h *[13] and *Sox2 *[14] regulate embryonic development prior to the organ formation. Later, *Brg1 *and *Snf2h *are thought to regulate retinal [15] and lens development [16], and *Sox2 *is required for lens placode formation [17] and optic cup formation [18]. AP-2α [19,20], pRb [21] and its partners, the E2Fs [22] play roles in both retinal and lens differentiation.

Rybp (RING1 and YY1 binding protein) is an evolutionarily conserved protein with a zinc-finger motive that was identified first as an interacting partner for the Polycomb group protein Ring1A [23]. Polycomb group (PcG) proteins function as transcriptional repressors acting part through histone modification, and are believed to be important regulators of organogenesis and cell lineage specification [24,25]. Recent studies in *Drosophila *have shown that *Drosophila *RYBP depends upon PcG proteins to repress transcription, suggesting that Rybp could be classified as a PcG protein itself [26]. Other studies have demonstrated Rybp's interaction with DNA binding transcription factors [27-29] as well as with apoptotic [30] and ubiquitinated proteins [31]. Although the precise molecular function of Rybp is not yet known, these interactions suggest that Rybp may be a multifunctional developmental regulator. Indeed, we have shown recently that the *Rybp *is required both for early mouse development and for proper brain formation. Specifically a dose-dependent role in the central nervous system (CNS) for *Rybp *was uncovered: haploinsufficiency in the subset of embryos caused an exencephalic phenotype due to imperfect closure of the neural tube [32].

Given established parallels between brain and eye development, we hypothesized that *Rybp *may also play a role in ocular development. Here we determined Rybp protein localization patterns in the murine eye and analyzed the function of *Rybp *in four mouse models representing reduced or increased *Rybp *gene dosage. These studies have shown that aberration in the normal protein levels of Rybp can result in retinal coloboma, abnormal lens and anterior eye development, and corneal neovascularization.

## Results

### Rybp is expressed in multiple tissues of the mouse embryonic eye

**Figure 1:**
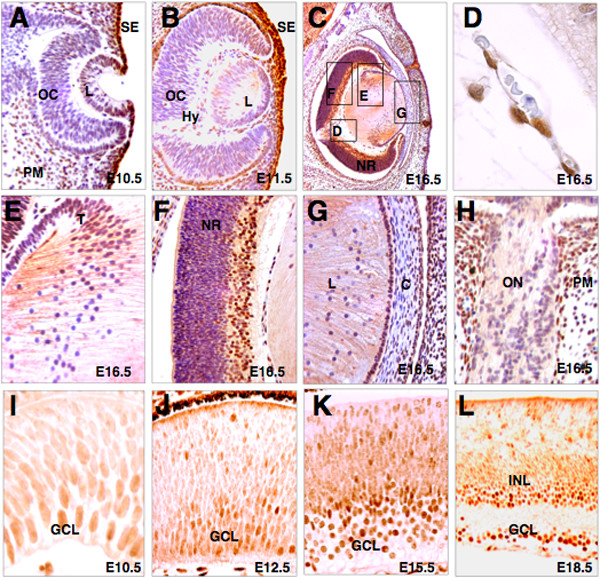
**Rybp localization during prenatal mouse ocular development**. (A-H) Sagittal sections were immunostained for Rybp (brown) and counterstained lightly with hematoxylin (purple) at E10.5 (A), E11.5 (B) and E16.5 (C-H). Higher-magnification of areas stained with the Rybp antibody indicated in (C) are shown in panels D-G. (I-L) Panels show the gradual increase of Rybp expression in the developing neural retina at E10.5 (I), E11.5 (J), E13.5 (K) and E18.5 (L). C; cornea, E; embryonic, GCL; ganglion cell layer, INL; inner nuclear layer, L; lens, NR; neuroretina, ON; optic nerve, PM; periocular mesenchyme, SE; surface ectoderm, OC; optic cup. Magnifications: A (×460); B(×320); C(×250); D(×800); E-H(×400); I(×630); J(×460), K(×320); L(×250).

In previous work, we reported the localization of Rybp in the developing CNS [32]. The common embryonic origin of the brain, lens, and retina from the primitive ectoderm prompted us to determine the protein localization of Rybp during mouse ocular development (Fig. 1). At E10.5, Rybp staining first strongly marked the head surface ectoderm surrounding the invaginating lens placode (Fig. 1A). Weak, speckled expression was also seen beneath the surface ectoderm, in the periocular mesenchyme, and throughout the emerging optic cup (Fig. 1A). From E11.5, Rybp appeared both in the anterior cells of the lens vesicle and in the nuclei of the elongating primary lens fiber cells (Fig. 1B). Similar to the lens, speckled expression was also evident in the optic cup, with a higher density of positive cells towards the ventral and marginal portion (Fig. 1B). Expression of Rybp in the hyaloid plexus was also detected (Fig. 1B). With progressive development, expression of Rybp persisted in the cells of the hyaloid cavity (Fig. 1C, D) and the head surface ectoderm. Between E14.5 and E16.5, Rybp localized in the differentiating secondary fiber cells of lens (E16.5; Fig. 1C and 1E) and in the ventral part of the neuroretina (E16.5; Fig. 1C, F). At E16.5 Rybp was also detected in the lens epithelium (Fig. 1C, G). In the cornea, Rybp was expressed in the corneal epithelium and some cells of the corneal stroma (Fig. 1G). Rybp displayed non-uniform weak staining in the cells of the optic nerve and stronger staining in the perioptic mesenchyme (Fig. 1H). One marked change in the expression pattern of Rybp during mouse eye development is that its widespread expression in undifferentiated cells of the retina becomes restricted to a layer-specific expression profile marking the gradually emerging ganglion (GCL) and inner nuclear (INL) cell layers (Fig. 1I–L). Rybp's expression is robust in the ganglion cell layer of the differentiating retina, but strong staining is also visible in the INL and in a few cells of the future photoreceptor layer of the neuroretina (Fig. 1L).

**Figure 2:**
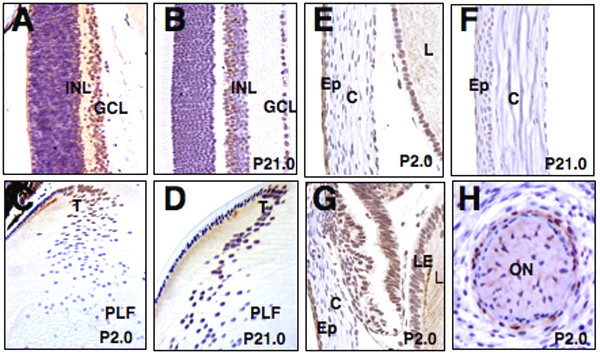
**Rybp localization in the postnatal mouse eye**. Sagittal sections of the eye at the level of the optic nerve at P2.0 (A, C, E, G, and H), and P21 (B, D, and F) were immunostained for Rybp. Shown are the retina (A, B), lens (C, D) cornea (E, F), connectiva (G) and optic nerve (H). C; cornea, Ep; corneal epithelium, GCL; ganglion cell layer, INL; inner nuclear layer, L; lens, LE; lens epithelium, ON; optic nerve, PLF primary lens fiber cells, T; transitional zone P; postnatal day. Magnification: (320×)

Next, we analyzed the localization of Rybp in two day (P2, Figs. 2A,C,E,G), and twenty one day old (P21, Figs. 2B,D) mouse eyes. In the postnatal P2 retina, Rybp showed intense staining in the GCL and in the differentiating INL of the retina (Fig. 2A). At P21, Rybp still was expressed in the GCL and in the INL, specifically in its dorsal part likely coincident with the bipolar and horizontal cell layers (Fig. 2B). Although Rybp was detected in the early stages of primary lens fiber cell development (Fig. 1B), in the more mature lens its expression was attenuated both in fully elongated primary lens fiber cells and in the lens epithelium (Figs. 2C and 2D). However, strong expression of Rybp in the lens was seen in the transitional zone where the secondary lens fiber cells are formed (Figs. 2C and 2D). At P2.0, Rybp was expressed in the majority of lens epithelial cells, and sporadic expression also was observed in the corneal epithelium, stroma, and basal membrane (Fig. 2E). By P21.0, Rybp is no longer expressed in the lens epithelium but still is weakly expressed in the corneal epithelium (Fig. 2F). Finally, Rybp was strongly expressed postnatally in the connectiva (Fig. 2G), and uniform localization of Rybp was observed around the newborn optic nerve (Fig. 2H). This dynamic and cell-type restricted expression pattern of Rybp (Figs. 1 and 2) raises the possibility that Rybp may have specific functional roles in the generation/maintenance of particular cell types during mammalian eye development.

### A subset of the *Rybp+/- *embryos exhibits retinal coloboma

**Figure 3:**
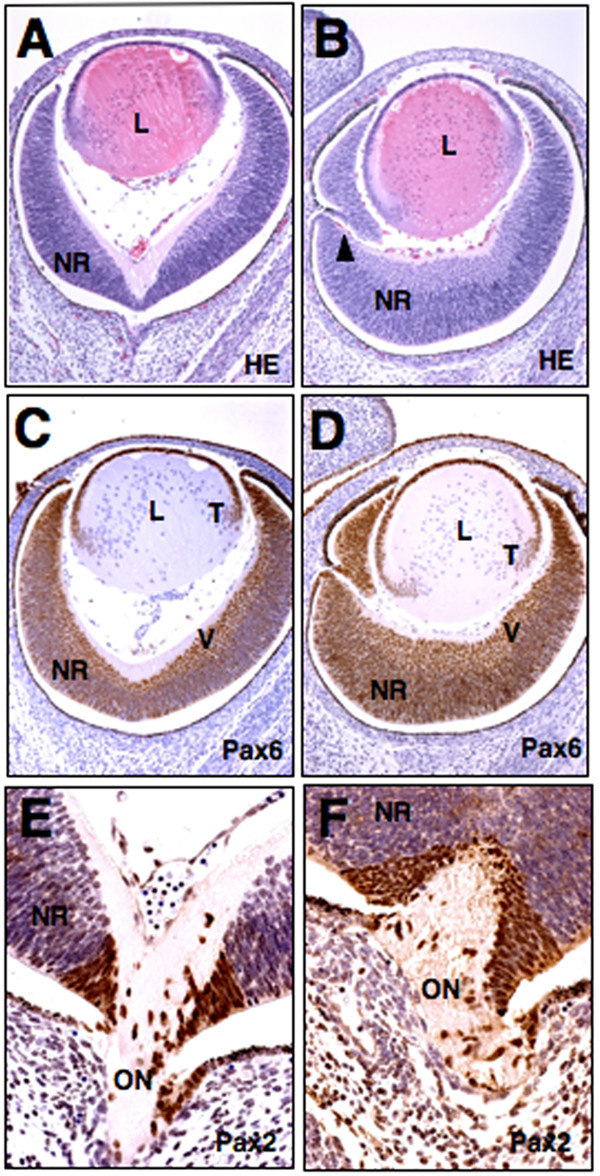
**Retinal coloboma in *Rybp*+/- mouse embryo**. (A-B) Hematoxylin and eosin- stained coronal sections of normal (A) and Rybp heterozygous null (B) eyes at E14.5. The neuroretina of the mutant eye is thickened and fails to close leading to the formation of coloboma (B; arrowhead). (C-D) Immunolocalization of Pax6 in wild type (C) and mutant (D) eyes. Pax6 is normally expressed in the ventral side of neuroretina. In the mutant eyes it shows broader expression in the retina and it is also more posteriorly positioned in the transition zone of the lens (C compare to D). (E-F) Immunolocalization of Pax2 in wild type (E) and mutant (F) eyes. L; lens, NR; neuroretina, ON; optic nerve, V; ventral, T; transitional zone. HE; hematoxylin and eosin. Magnifications: A-D(×320); E-F(×460)

Previously we reported that a subset of *Rybp *heterozygous null embryos exhibited perturbed brain development including forebrain overgrowth and exencephaly [32]. As the retina forms from the forebrain, we next examined the possibility of aberrant eye development in these animals. We found that 32% (6/19) of *Rybp*+/- exencephalic mice examined at several stages of development (from E12.5 to postnatal stages) showed retinal/optic nerve coloboma (compare Fig. 3A and 3B). Colobomas of this type often are caused by an incomplete closure of the optic fissure that occurs normally at E13.5 [33]. *Rybp*+/- colobomas were observed both bilaterally and unilaterally. In addition, *Rybp*+/- eyes had thickened neuroretinas, their lenses were ventrally rotated and misplaced within the eyeball (see Fig. 3B in comparison to Fig. 3A). The optic nerve was also frequently regressed (Fig. 3F). One possible explanation for the development of colobomas is that the decreased level of Rybp in the mutant retinas influences the normal distribution of regulatory proteins such as Pax6 [34-36] or Pax2 [33,37,38]. Both *Pax6 *and *Pax2 *have been shown to be essential for proper closure of the optic fissure. Accordingly, we tested whether immuno-localization of Pax6 and Pax2 was affected in the mutant *Rybp *eyes. Normally, Pax6 protein is localized in the ventral side of the retina but disappears from the developing optic nerve after E12.0 in wild-type animals [39]. In the *Rybp *mutant embryos, Pax6 expression spreads across the entire thickness of the retina, expanding to its margin (Figs. 3C, D). In contrast, the localization of Pax2 in mutant eyes were unchanged (Fig. 3E compare to 3F).

**Figure 4:**
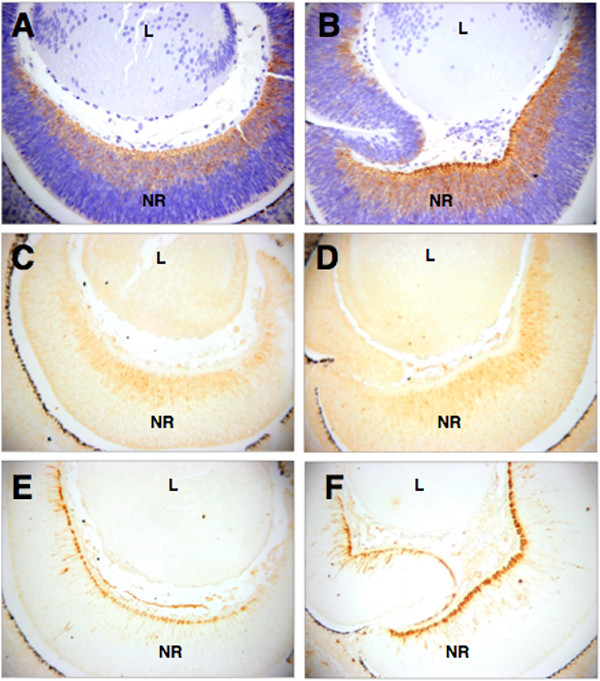
**Progenitor cell fate is not changed in the mutant retinas of the *Rybp *heterozygous eyes**. Wild type (A, C, E) and *Rybp *heterozygous null eyes exhibiting retinal coloboma (B, D, F) were stained with TUJ1 (A, B), NeuN (C, D) and Nestin (E, F) at E14.5. (A-B) TUJ1 staining, marking early neuronal cell types including ganglion cells of the retina, is comparable in the wildtype and mutant mice. (C-D) Similarly, NeuN, a postmitotic neural marker, shows no significant alteration in the mutant retinas. (E-F) The distribution of nestin, an intermediate filament marker for neural progenitor cells, is not affected in the mutant retinas. L; lens, NR; neuroretina, Magnifications: (×460)

The proper ratio between neural progenitor and postmitotic neuronal cell types is important for normal retinal development and disturbed morphogenesis of this process can lead to colobomas. [40]. Accordingly, we investigated whether the ratio between early- and late-born neurons changed in the mutant retinas exhibiting the coloboma phenotype. In the prenatal mutant retina, expression of specific neuronal cell fate markers was similar to the expression of these markers in the retina of control mice. These included Tuj1 (marks early neural cell types; Fig. 4A,B), NeuN (postmitotic, marks late neuronal cell types; Fig. 4C, D) and nestin (marks neural progenitor cells; Fig. 4E, F). The apparent normal distribution of these neuronal markers shows that the neuronal cell differentiation is not affected in the *Rybp *mutant retinas and probably is not the direct cause of the failure of optic fissure closure.

### Rybp-/- <-> Rybp+/+ chimeric embryos show a series of eye defects

**Figure 5:**
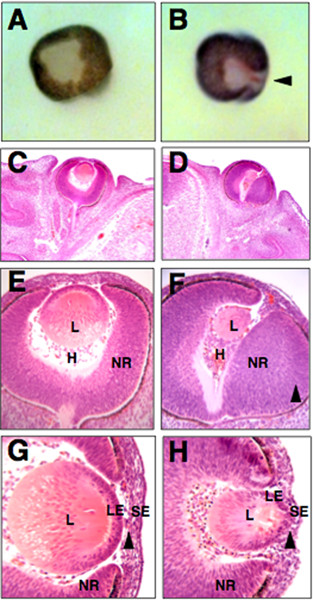
**Multiple ocular abnormalities in *Rybp-/- <-> Rybp+/+ *chimeric embryos**. (A-B) Whole mount eyes from E13.5 wild type (A) and chimeric (B) embryos. The normal eye is symmetrical, while in the chimeric eye the chorioid fissure fails to close, leaving a large coloboma (arrowhead). (C-F) Histology of coronal sections of E13.5 wild type (C, E) and chimeric (D, F) eyes. Higher-magnification views of areas indicated in (C) and (D) are shown in (E) and (F), respectively. In the chimeric embryos, the eyes are rotated, lens development is delayed and overall lens size is reduced, and the retina is asymmetric with thickening on one side (F; arrowhead). (G-H) At E13.5 in the normal eye there is a mesenchymal cell layer between the SE and the LE (G; black arrowhead). In the chimeric eye, the lens epithelium is continuous and mixed with the mesenchyme and the surface epithelium (H; black arrowhead). H; hyaloid cavity, L; lens, LE; lens epithelium, NR; neuroretina, SE; surface ectoderm. Magnification: A-B (×120); C-D (×60); E-F (160×); G-H (×400)

Early lethality of *Rybp -/- *embryos [32] prevented our analysis of the effect of a complete loss of Rybp during eye development. However, chimeric mice (n = 90) have been generated from *Rybp *homozygous null -/- and wild type (+/+) ES cells, and 20% of them showed low overall contribution of *Rybp -/- *ES cells and displayed forebrain abnormalities [32]. Half of the chimeric embryos (examined between E9.5 and E14.5) with brain abnormalities also showed eye defects similar to those described above for the *Rybp*+/- embryos. These included retinal colobomas (Figs. 5A and 5B), and defects in lens formation (compare Figs. 5D to 5C and 5F to 5E). In addition, in an E13.5 chimera, the separation between the lens epithelium and the surface ectoderm was compromised (compare Fig. 5H to 5G). Our findings again indicate that normal lens and retinal development cannot occur in the context of suboptimal Rybp dosage.

### Overexpression of *Rybp *results in lens, retinal and corneal defects

**Figure 6:**
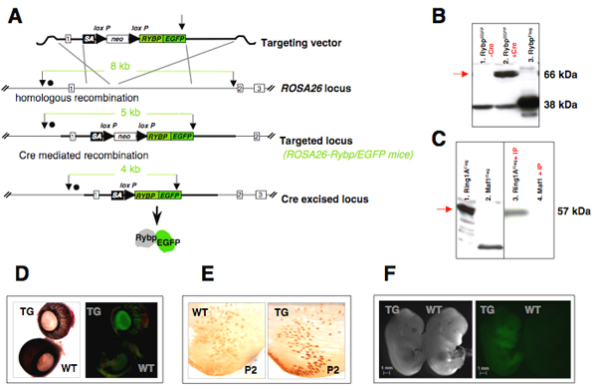
**Lens-specific and ubiquitous expression of RYBP/EGFP fusion protein**. (A) Scheme illustrating the strategy used to generate a Cre recombinase-mediated conditional ectopic *Rybp *allele by targeting the *ROSA26 *locus (for details, see Methods). The position of the hybridization probe (dot) and restriction enzyme EcoRV (arrows) used for detecting the correct homologous integrants by Southern blotting is shown. The 5' probe used detects an 11 kb wt band and a 3.8 kb targeted band, due to the presence of an extra EcoRV site in the targeted allele. The exons shown as open boxes. Cre: Cre-recombinase, EGFP; enhance green fluorescent protein, Neo; neomycin phosphotransferase. (B) Western blotting analysis showing the expression of the RYBP/EGFP fusion in lysates of cells that have been exposed to Cre recombinase. Lane 1, lysates from *ROSA26 *knock-in ES cells prior to Cre exposure (-Cre); lane 2, lysates from *Rosa26 *knock-in ES cells after Cre exposure (+Cre); lane 3, lysates from ES cells transfected with an Rybp^Flag ^construct, as a positive control. The blot was probed with the anti-Rybp antibody. (C) Ectopically expressed *Rybp *can associate with Ring1A. Lanes 1–2: Cells lysates of transfected ES cells blotted with anti-Flag antibody showing that both Ring1a^Flag ^(Lane 1) and Maf1^Flag ^(Lane 2) strongly expressed in the transfected cells; Lane 1, Cells transfected with *Ring1A*^*Flag *^construct., Lane 2, Cells transfected with *Maf1*^*Flag *^construct. Lane 3–4: Cells transfected with either *Ring1A*^*Flag *^(Lane 3) or *with MAF1*^*Flag *^(Lane 4), then immunoprecipitated with Rybp and blotted with anti-Flag antibody. Lane 3 shows the 57 kdDa Ring1A^Flag ^fusion protein as the result of the association with the RYBP/EGFP fusion protein. Maf1 cannot bind Rybp (Lane 4). (D) P1 eyes harvested from double transgenic (*ROSA26-RYBP/EGFP; αA-crystallin/Cre *mice) and wildtype (WT) animals shown in dark field and fluorescence. (E) Lens specific over-expression of the RYBP/EGFP fusion protein in newborn (P2) lenses. EGFP immunohistochemistry shows the overexpression of the transgene in the *ROSA26-RYBP/EGFP; αA-crystallin/Cre *lens (TG). The wild-type lens shows only background staining (WT). (F) Bright-field and dark-field pictures of E11.5 littermates. All embryos that genotyped double transgenic for *ROSA26-RYBP/EGFP;β-Actin/Cre *also exhibited detectable EGFP fluorescence (TG) when compared to wildtype (WT) littermates. Magnification: D (×40); E (×10)

Next we employed a conditional ectopic overexpression strategy [41] to assess the effects of *Rybp *overexpression in the lens. First we introduced an inducible Rybp – green fluorescent protein (EGFP) fusion gene into the mouse genome (*ROSA26-RYBP/EGFP *mice; Fig. 6A). Proteins were extracted from the targeted ES cell lines (*ROSA26-RYBP/EGFP *cell line) prior to and following Cre induction (*ROSA26-RYBP/EGFP; Cre *cell line), and the expected 66 kDa Rybp/EGFP fusion protein was observed (Fig. 6B). To demonstrate that this fusion protein is functional and can bind to Ring1A as described earlier [23], the excised cells (*ROSA26-RYBP/EGFP; Cre *cell line) were transiently transfected with Flag-tagged Ring1A, and lysates were immunoprecipitated with either RYBP or GFP antibodies and blotted with a Flag antibody. As expected, the RYBP/EGFP fusion protein was found together with Ring1A *in vivo *(Fig. 6C).

**Figure 7:**
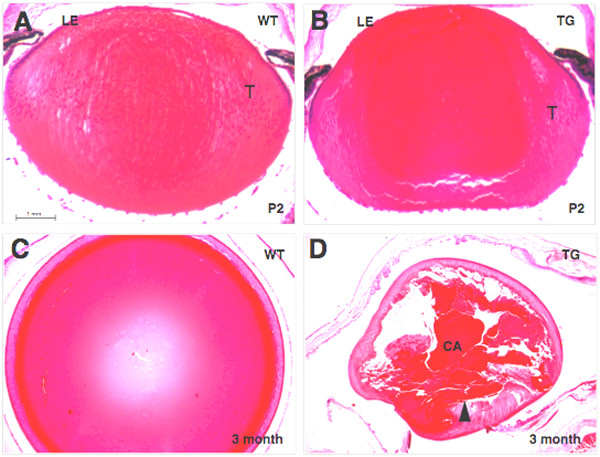
**Abnormal lens development in the lens-specific *Rybp *transgenic mice**. (A-D) Histological appearance of wild type (A, C) and *ROSA26-RYBP/EGFP; αA-crystallin/Cre *transgenic (B, D) lenses at early postnatal (P2) development (A, B) and adulthood (3 month) (C, D). (A, C) Wild type eyes with normal morphology of the lens epithelium and fiber cells. (B, D) Transgenic eyes showing cortical inhomogeneity as a sign of developing cataract and impaired lens development (B). Lenses of adult transgenic mice (3 month) show progressed cataractous morphology (D). CA; cataract, L; lens, LE; lens epithelium, T; transitional zone, TG; transgenic, WT; wild type; P; postnatal. Magnification: A-B (×160); C-D (×250); E-F (×120)

**Figure 8:**
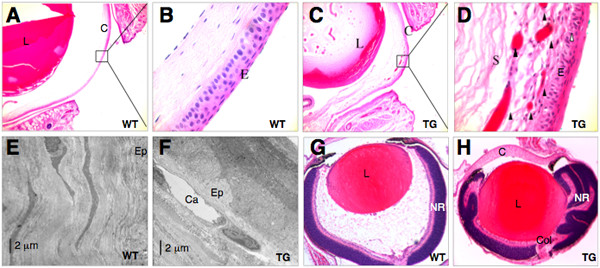
**Corneal neovascularization and irregular retinal development in ubiquitous *Rybp *transgenic mice**. (A-F) Neovascularization of the *ROSA26-RYBP/EGFP;β-Actin/Cre *transgenic corneas in adult (2-month old) mice. Sagittal sections from wild type (A, B) and transgenic eyes (C, D) were stained with hematoxylin and eosin; boxes in panels A and C show areas magnified in panels B and D, respectively. The appearance of vessels (arrowheads in panel D) in the stroma of transgenic mice only is indicative of corneal neovascularization. The epithelium of the transgenic mice is also disorganized (D; open arrowhead). (E-F) Electron micrographs showing the corneal stroma next to the corneal epithelium in transgenic (Tg) and wild-type (Wt) animals at three weeks of age. While the stroma of the wild-type animal is not vascularized, a capillary (Ca) is seen close to the corneal epithelium in the transgenic animal. (G-H) Abnormal retinal folding and colobomas in newborn *β-Actin/RYBP *Tg mice (H) in comparison to normal retina in controls (G). C; cornea, Col; coloboma, E; epithelium, Ep; corneal epithelium; L; lens, NR; neuroretina, WT; wild type, S; stroma, TG; Transgenic. Magnification: A, C (×200); B, D (×460); G-H (×160)

The *ROSA26-RYBP/EGFP *mice were crossed with two different reporter mouse lines and the proper expression of the fusion protein was confirmed by fluorescent microscopy: lens specific expression was seen for the *ROSA26-RYBP/EGFP;αA-crystallin/Cre *double transgenics (Fig. 6D) and ubiquitous expression for the *ROSA26-RYBP/EGFP;β-Actin/Cre *double transgenics (Fig 6F). Aberrant lens morphology of the *ROSA26-RYBP/EGFP; αA-crystallin/Cre *mice is shown in Fig. 7. Although the P2 embryonic lenses showed only subtle abnormalities in fiber cell morphology (7A–B), older mice developed severe opacities of the lens resulting in a collapse of lens fiber mass (Figs. 7C–D). Eyes from *ROSA26-RYBP/EGFP; β-Actin/Cre *mice were examined at embryonic (E16.5, E18.5), postnatal (P1–P4, P7, P14, P21) and adult stages (2, 3, 6 month) (Fig. 8, and data not shown). This ubiquitous overexpression of RYBP/EGFP led to the abnormal formation of a number of ocular tissues. The most frequent phenotype seen (in 35% of the mice) was neovascularization of the corneas (compare Fig. 8C–D to 8A–B). These structural changes were visible at postnatal stages (P7-21) during which the stromal layer thickened. When hemizygous mice were mated to obtain mice homozygous for the *RYBP/EGFP *transgene, the penetrance of the phenotype increased to 80%. Small vessels were apparent in the stroma of the cornea under bright field microscopy, and by 2–4 months postnatal, gross corneal neovascularization was visible (data not shown). Electron microscopy studies showed that the capillaries are already present at birth in the transgenic corneas (Fig. 8E, F). This was further supported by immunohistochemistry using an anti-CD34 antibody which marked the newly formed vessels of the mutant corneas (data not shown). Aside from neovascularization, other observed ocular phenotypes included irregular folding of the retina, retinal coloboma, defects in anterior eye development (absence of the vitreous body, absence of the anterior chamber), and lens opacification during later adulthood (Fig. 8G,H and data not shown). In summary we have developed transgenic mouse models in which expression of *Rybp *in the lens disrupted normal fiber cell differentiation and ubiquitous expression of *Rybp *resulted in corneal neovascularization and defects in the anterior eye development. These models suggest that normal eye development is sensitive for the proper dose of *Rybp *and that improper dosage of *Rybp *causes multiple eye abnormalities.

### Genes with altered expression level in the *Rybp *transgenic lenses

**Figure 9:**
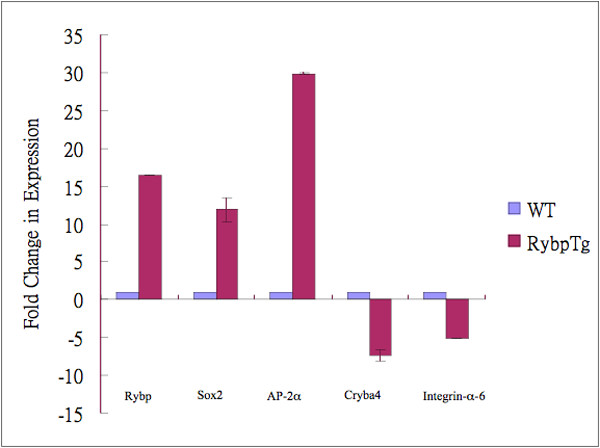
**Quantitative RT-PCR analysis of transgenic lenses overexpressing *Rybp***. Relative change in expression level *of Rybp*, *Sox2*, *AP-2α*, *βA4-crystallin *and *α6-Integrin *transcripts in the P1 lens. All data are normalized to *B2M *expression level. Error bars indicate standard deviation.

As a first attempt to elucidate the molecular basis of abnormal lens fiber cell differentiation of the *ROSA26-RYBP/EGFP; αA-crystallin/Cre *mice (Figs 7, 8), we performed expression analysis of the genes involved in this process including major lens structural proteins, selected cell adhesion molecules, and major transcription factors implicated in lens development (Fig. 9). Total RNA from P1 transgenic and control lenses was used to synthesize cDNA, and quantitative RT-PCR was performed. The expression level of both *Rybp *and *EGFP *mRNA was increased 16-times compared to the non-transgenic control as expected. From the major lens structural proteins, the *Cryba4 *transcript showed a seven-fold reduction in transgenic lenses, while the expression of other *crystallins *and *filensin *was unchanged. From the tested cell adhesion molecules, *α6-integrin *mRNA level was five-times reduced while other *integrins (α5- and β1-integrins*) remained unchanged. Among tested transcription factors, *AP-2α *mRNA level was 30-times and Sox2 was 12-times elevated. The level of transcription factors *Pax6*, *Prox1*, *MafA*, *MafB *and *c-Maf *did not change significantly in the *ROSA26-RYBP/EGFP; αA-crystallin/Cre *lenses. These results further confirm that normal lens development depend on the *Rybp *gene dosage. RNA microarray studies may need to be conducted in the future to provide further insight into which membrane proteins, gap junctions and β/γ-crystallins are being affected.

## Discussion

*Rybp *encodes an essential regulatory protein involved in early post-implantation development of the mouse embryo. In addition, *Rybp *plays important roles in organogenesis as evidenced by disrupted development of the forebrain found both in *Rybp *heterozygous null and chimeric embryos [32]. Here we characterized the protein localization and *in vivo *function of *Rybp *in another organ – the developing mouse eye. We show that *Rybp *is required for normal retinal and lens development, and may also be involved in the formation of the anterior eye segment.

Our previous work revealed that during CNS development, Rybp is predominantly localized in postmitotic neurons and differentiated cell types of the developing mouse embryo [32]. In the present study, we show that in the developing mouse eye, Rybp also becomes robustly made in the differentiating layers of the neuroretina including the ganglion and inner nuclear layer cells. This suggests once again a possible role for *Rybp *in cell cycle exit or the commitment to differentiation. Gaining further insight into the precise cellular role of *Rybp *during mouse ocular development likely requires a conditional mutant version of the gene where *Rybp *can be eliminated in specific cell lineages or at specific time points of embryonic development.

The major phenotypic malformation observed in all *Rybp *mouse models (heterozygous, chimeric and transgenic, see Table 1) was the failure of the closure of the optic fissure, retinal coloboma. Morphological analysis of embryonic eyes showed that 32% of *Rybp *deficient (heterozygous) and 50% of chimeric mice have eye coloboma in conjunction with brain defects, but coloboma was also seen in the *Rybp *transgenic mice that did not display obvious brain defects. A number of mammalian genes encoding transcription factors (*BCOR*, *CBP*, *Chx10*, *Cited2*, *c-Maf*, *Foxg1*, *Pax2*, *Pax6*, *Ptch*, *Six3*, *Ski*, *Vax1 and Vax2*), signaling molecules (*Jnk1*, *Jnk2 and Shh*), or members of the retinoic acid pathway have been associated with coloboma [5]. Our study describes *Rybp *as a novel gene associated with coloboma. While the molecular and cellular mechanisms underlying this condition are still poorly understood, they likely involve perturbations in cell adhesion, cell shape, cell proliferation, cell death, and/or the extracellular matrix.

**Table 1:** Summary of ocular phenotypes

**Ocular phenotype**	**Examined Mutant**	**No. with ocular phenotype/No. of examined mutants**	**Penetrance (%)**
**Coloboma **(by E18.5)	*Rybp *heterozygous (+/-) mice	5/15	33
**Coloboma**, lens defects (By E14.5)	Chimeric mice *Rybp-/- <->*	6 exencephalic/19	32
	*Rybp+/+*	3 non exencephalic/19	16
**Cataract **(by 3 month)	*ROSA26-Rybp/EGFP; αA-crystallin/Cre Tg/+ *mice	44/75	59
**Neovascularization **(by 3 month)	*ROSA26-Rybp/EGFP; β-Actin/Cre Tg/+ mice*	30/87	35
**Neovascularization **(by 3 month)	*ROSA26-Rybp/EGFP; β-Actin/Cre Tg/Tg mice*	30/38	80
**Coloboma, irregular retinal folds, anterior chamber defects **(by P7)	*ROSA26-Rybp/EGFP; β-Actin/Cre Tg/Tg mice*	11/38	29

Aside from the retinal colobomas, a wide range of lens abnormalities were found in the Rybp mouse models. In both *Rybp *heterozygous and chimeric eyes, ventral rotation of the lens was found in association with coloboma (see Fig. 3 and 5). A similar phenotype has been reported for *Otx *mutant mice [42]. Lenses of *Rybp *chimeric embryos showed abnormal separation of the lens vesicle from the surface ectoderm (Fig. 5), a condition found in Pax6 heterozygous [43] and Foxe3 homozygous [44] mouse embryos. Transgenic lenses overexpressing Rybp developed abnormally differentiated lenses at birth (if not earlier), and this condition further deteriorated with age resulting in a total collapse of the lens fiber mass (see Fig. 7). This demonstrates that high level of Rybp disrupts normal fiber cell differentiation and maturation. Similar phenotype was observed when *AP-2α *transcription factor, individual *E2F*s or growth factors were overexpressed in lens specific manner ([19,45], see review [46]). Whether excess of Rybp interferes with adhesion and migration (like AP-2α), cell cycle (like E2Fs), terminal differentiation (like TGFβ) of secondary fiber cells or acts via completely different mechanisms, requires further studies. Indeed, *RYBP *was shown to be involved in specification of function within the family of *E2F *transcription factors in cell culture systems [28] however this has not been investigated *in vivo *during lens fiber cell differentiation.

Ubiquitous overexpression of *Rybp *caused similar lens defects and additionally led to corneal neovascularization (see Fig. 8). Genes such as *VEGF, FGF2 *and *MMP-2 *have already been associated with corneal neovascularization [47-50]. A possible connection between *Rybp *and angiogenesis warrants further investigation, especially in light of the observation that during embryonic development *Rybp *is expressed in hyaloid vessels and in the endothelium of blood vessels outside of the eye (Fig. 1; Pirity and Schreiber-Agus, unpublished data).

The variable penetrance of the phenotypes may indicate that expression and/or function of the retained wild type *Rybp *allele is being modified differentially in affected or non-affected animals. This is also supported by our previous observation that the penetrance of the exencephaly, a previously described heterozygous semipenetrant phenotype, was influenced by genetic background [32]. Indeed the phenomenon "semi-penetrance" is a common observation in genetically-engineered mice.

One potential molecular mechanism for abnormal eye development in *Rybp *mice could relate to Rybp's role in Polycomb group protein transcriptional regulation [23]. Polycomb proteins function in multiprotein complexes to regulate expression of homeotic and other regulatory genes during embryonic development [51-57] and also in eye development [58,59]. Notably, in our limited gene expression studies using a candidate approach (Figure 9), several key eye regulators were found to be significantly affected in the *Rybp *transgenic lenses examined, including Sox2 and AP-2α (see also [60,18,19,61]) as well as were some of the crystallins [62]. Moreover, Pax6 localization in retina and lens appears affected in the *Rybp *heterozygous null mice (Figure 3). Taken together, our findings suggest that *Rybp *may be regulating the expression of other developmental regulators, whose altered levels in the *Rybp *mouse models could be causal to the observed phenotypes. Further studies are necessary to determine whether *Rybp *directly regulates these (and other) genes, and whether this regulation occurs via the interaction of *Rybp *with Polycomb group transcriptional repressors. Interestingly, the abnormal anterior eye formation of *Ring1*-deficient mice, which is exacerbated in compound *YY1+/-Ring1-/- *mice, is similar to what we have observed in the *Rybp *mutant mice ([59] and Fig. 7H). Since both YY1 and Ring1A interact with Rybp and with other class II PcG proteins, it is possible that these proteins functionally interact to orchestrate normal vertebral eye development.

## Conclusion

Collectively, the present work provides the first *in vivo *evidence for a role of the Polycomb group binding protein Rybp in mouse eye development and disease. Further studies need to address the molecular basis of this role and to determine how Rybp functionally relates to other known regulators of ocular processes.

## Methods

### Es cell injections, mouse breeding, husbandry and genotyping

ES cell injections, mouse breeding, husbandry, and genotyping of the Rybp knockout colonies was as previously described [32]. Chimeric embryos were generated by microinjecting *Rybp*-/- R1 ES cells (129/Sv × 129-Cp; [63]) into blastocysts derived from wild-type (*Rybp+/+*) mice (C56/BL6), as described previously [32]. The *αA-crystallin/Cre *transgenic mice were a kind gift of M.L. Robinson (Columbus, Ohio, USA); [64]. Analysis of the Cre-mediated recombination pattern in the αA-Cre line was performed by mating with the *ROSA26 *reporter line (*Gt(ROSA)26Sor*^*tm*1*Sor*^) as described [65]. The *β-Actin/Cre *transgenic mice (*FVB/N-Tg(ACTB-cre)2Mrt/J*) line *MRL *[66] were purchased from Jackson laboratories (Bar Harbor, Maine, USA). *Cre *mice were crossed with the *Rybp *transgenic mice (see next) to obtain F1 *Rybp *transgenic mice (FVB).

Mice were housed in a 12-h light, 12-h dark cycle and maintained in the animal facility at AECOM in accordance with institutional guidelines. Noon of the day the vaginal plug was observed was considered E0.5 of embryogenesis. For genotyping targeted ES cell colonies/mice, *EGFP *Primers: A, (5'-aagttcatctgcaccaccg-3') and B, (5'-tgctcaggtagtggttgtcg-3') were used as described. The genotypes were determined by PCR analysis on DNA extracted from the tail or yolk sac.

### *Rybp *conditional transgenics

The strategy used to generate a Cre recombinase-mediated conditional ectopic *Rybp *allele by targeting the ubiquitous *ROSA26 *locus is shown on Figure 6. The *ROSA26 *locus (referred to as an R26 knock-in) was targeted with a floxed neo cassette followed by an RYBP/EGFP fusion. To generate the RYBP/EGFP fusion, the Rybp open reading frame (ORF) was amplified with primers A (5'-gcacgtcgaccagcccgtccatgaccatgg-3') and B (5'-ctctggatccgaaagattcatcattcactgc 3') and cloned into *pEGFP-N3 *with NheI/SalI. The Rybp/EGFP fusion was transferred to pBigT [41] with NheI/Not1, and then the Rybp/EGFP together with the floxed neomycin cassette was cloned into the *pROSA26 *vector [41] with AscI/PacI. *Rybp *transgenic ES cell lines were generated by electroporating linearized targeting vector into R1 ES cells [63] as described in 96 clones resistant to G418 (Gibco, 300 μg/ml) were selected, and screened by genomic Southern blot hybridization on DNA digested with EcoRV as previously described [41]. Two targeted clones were injected into C57BL/6 blastocysts and produced germ-line chimeras (*ROSA26-RYBP/EGFP *mice) [67]; mice carrying the targeted allele were genotyped by the PCR as described [41]. Male chimeras were mated with ICR females, and their agouti offspring were tested for transmission by tail PCR and blotting. Animals heterozygous for the mutation were bred with corresponding *Cre *transgenic lines and analyzed for the phenotype. Non-transgenic littermates were used as controls for all experiments. Heterozygous transgenic progeny were mated to maintain the allele. All analyses were performed on a mixed (129 × ICR) background and mutant mice were analyzed in comparison to their wild-type littermates.

### Histology and immunohistochemistry

All embryos were dissected in PBS, fixed overnight in PBS buffered 4% paraformaldehyde, and paraffin-embedded sections (6 μm) were mounted for staining. For immunohistochemistry, deparaffinized and rehydrated tissue slides were first treated for 30 min with 3% H_2_O_2 _to inactivate endogenous peroxidases. After rinsing washing in PBS for 5–10 min, slides were blocked with 10% (w/v) BSA in PBS and then incubated overnight at 4°C with antibodies against Rybp (anti-DEDAF; dilution 1:1000; Chemicon, AB3637; rabbit), Pax2 (dilution 1:200, Babco, PRB276P, rabbit), Pax6 (dilution 1:500, mouse IgG1, DSHB), Nestin (dilution: 1:100, mouse IgG1, DSHB Rat-401), NeuN (dilution: 1:1000, Chemicon MAB377), or TUJ1 (dilution: 1:2000, Sigma T8660). After removing excess antibody, samples were incubated with a 1:400 dilution of biotin-conjugated secondary anti-rabbit (Dako, EO466) or anti-mouse antibodies (Dako, EO433) for 45 minutes at room temperature, washed in PBS, and incubated with avidin-biotinylated enzyme complex for 45 minutes. The reaction was developed with the DAB kit (Vector labs). For better visualization, slides were often slightly counterstained with hematoxylin. Samples were viewed and photographed under epifluorescent illumination with Leica MZFLIII microscope.

### Electron microscopy

Dissected P1 and P21 day old eyes were placed in Ito's fixative [68] for 24 h after the cornea had been pierced with a fine needle. The eyes were washed overnight in cacodylate buffer, postfixed with OsO4, dehydrated, and embedded in Epon (Roth, Karlsruhe, Germany). Semithin sections were stained with toluidine blue. Ultrathin sections were stained with uranyl acetate and lead citrate and viewed with a Zeiss (Oberkochen, Germany) EM 902 electron microscope.

### Western blots

Western blotting analysis was conducted upon protein extracts from embryonic stem cells as described earlier [32]. Primary antibodies were used against RYBP (anti-DEDAF; 1:1,000, Chemicon AB3637), GFP (1:1,000, Molecular Probes A11122) and Flag HRP (1:5000, M2, Sigma).

### Quantitative RT-PCR

Total RNA isolated from the lens and retina of the *ROSA26-Rybp/EGFP; αA-crystallin/Cre *P1 and P21 day old mice was extracted and reverse transcript was obtained as described elsewhere [16]. Real time PCR was performed as previously described using primers to amplify *Pax6, Prox1, MafA, MafB *and *c-Maf *transcripts [69,70]). The additional primers are: *Rybp *primers A, (5'-agaccagcgaaacaaaccac-3') and B, (5'-aggaggagcgagtcttttcc-3'); *Crystallin βA4 (Cryba4) *primers A, (5'-gggtttgttcccagttcct-3') and B, (5'-acctgagtggtgatcgctct-3'); *Filensin (Bfsp1) *Primers: A, (5'-cattgagattgaaggcagca-3') B, (5'-acactggatccaaggctgag-3'); *AP2α *primer A, (5'-gtgtcagagatgctgcggta-3') and B (5'-tgaggatggtgtccacgta-3'); *Integrin α-6 *primers A, (5'-attctcctgagggcttccat-3') and B, (5'-ttgagggaaacaccgtcact-3'); *Sox2 *primer A, (5'-acttttgtccgagaccgaga-3') and B, (5'-ctccggcaagcgtgtactta-3'); and *B2M *primers A, (5'-catacgcctgcagagttaagc-3') and B, (5'-gatgcttgatcacatgtctcg-3'). Amplification of the cDNA was performed using 7900 HP Applied Biosystems Real Time PCR machine. Relative fold changes were calculated using CCNI as an internal control as described [70].

## Abbreviations

C; cornea, Ca; capillary, CA; cataract, Co; cortex, Col; coloboma, Cre; Cre recombinase, E; embryonic day, Ep; corneal epithelium, GCL; ganglion cell layer, EGFP; enhanced green fluorescent protein, H; hyaloid cavity, HE; hematoxylin and eosin, INL; inner nuclear layer, L; lens, LE; lens epithelium, M; marginal, ME; mesenchyme, NE; neuroepithelium, NR; neuroretina, P; postnatal day, PLF; primary lens fiber cells, PcG; polycomb group protein; OC; optic cup, ON; optic nerve, ONL; outer nuclear layer, ORF; open reading frame, PCR; polymerase chain reaction, PM; periocular mesenchyme, qRT-PCR; quantitative reverse transcriptase – polymerase chain reaction; S; stroma, SE; surface ectoderm, T; transitional zone, V; ventral, TG; transgenic, WT; wild type

## Authors' contributions

M.K.P, N.S.A and A.C. conceived the project, designed the experiments, and wrote the manuscript. M.K.P. performed most of the experimental manipulations. W.W. carried out the RT-PCR analyses. L.V.W. contributed to the phenotypic analyses. E.T. performed the E.M. experiments and contributed to the phenotypic analyses. All authors read and modified drafts, and approved the final manuscript.
